# Approaches to Medication Administration in Patients With Lack of Insight

**DOI:** 10.7759/cureus.27143

**Published:** 2022-07-22

**Authors:** Okelue E Okobi, Ogochukwu Agazie, Oghenetega E Ayisire, Funmilola Babalola, Anthony I Dick, Zainab Akinsola, Adeyemi A Adeosun, Oluwasayo J Owolabi, Temitope O Ajayi, Adetayo Y Odueke

**Affiliations:** 1 Family Medicine, Lakeside Medical Center, Belle Glade, USA; 2 Medicine, College of Medicine University of Lagos, Idi Araba, NGA; 3 Psychiatry, University of South Wales, Pontypridd, GBR; 4 Epidemiology and Public Health, Texas Department of State Health Services, San Antonio, USA; 5 Public Health, Chicago State University, Chicago, USA; 6 Internal Medicine/Family Medicine, Windsor University School of Medicine, Toronto, CAN; 7 Molecular Pharmacology and Experiment Therapeutics, Mayo Clinic, Rochester, USA; 8 Psychiatry, Lugansk State Medical University, Lugansk, UKR; 9 Internal Medicine, University at Buffalo, New York, USA; 10 Medicine, Olabisi Onabanjo College of Health Sciences (OACHS), Lagos, NGA

**Keywords:** clinical competence, medication therapy management, psychiatry & mental health, insight, lack of insight

## Abstract

Lack of insight typically complicates psychiatric presentations, necessitating careful thought and planning to choose the best course of treatment. Exploring methods of medication administration techniques in the context of a lack of insight is crucial to achieving the ultimate goal of overcoming the insight barrier as rapidly as possible, which will result in therapeutic benefit. This study's objective was to systematically review the evidence on medication administration techniques in a backdrop of lack of insight and how that evidence was curated in the scientific literature. This study used the literature search strategy, which entails retrieving and analyzing the existing scientific literature pertinent to medication administration techniques for individuals with no insight between 2010 and 2022. Accessing online databases, such as PubMed, Google Scholar, and Medline was utilized in this study's literature search strategy. In our findings, in the primary evidence search, no randomized control trial (RCT) comparing the various models of medication administration with a lack of insight was found. No study provided data on the superiority of utility, quality of life, or efficacy outcome. Some 17 scientific papers were identified that cited various trials about lack of insight and medication use and met the inclusion criteria. We concluded that it could be challenging to administer medication to patients who lack insight.

Nonetheless, progress has been made to mitigate this obstacle. Common moral values, common sense, medicolegal support, person-centered integrated care, and cutting-edge medication techniques may play a role. However, these models of medication administration are still evolving, along with the ethical concerns accompanying them. Hopefully, the available models discussed in this analysis will serve as a foundation for future developments. Nonetheless, much remains to be done. We encourage contemporary research to investigate safer and more dynamic methods that can alleviate this condition.

## Introduction and background

Medication administration is one of the most important ways to treat most diseases. The benefit of pharmacological therapies and medication administration in psychiatric disorders have been weighed alongside numerous other psychiatric treatment options like somatic (physical) therapies, psychological therapies, milieu therapy, therapeutic community, activity therapy, psychopharmacology, electroconvulsive therapy, and psychosurgery [1-2], and has shown some degree of variation in therapeutic advantage over other modalities. The dangers associated with pharmacotherapies can arise from unintended consequences, such as treatment errors, underdosing or overdosing, compliance, wrong drugs, wrong timing, adverse reaction, etc. [3]. Medication administration requires a deep understanding of the drugs, including their pharmacokinetics, pharmacodynamics, pharmacogenomics, indications, possible side effects, adverse reactions, drug-drug interactions, and drug storage and inventory management [4]. To maximize the benefits of pharmacotherapy in psychiatry, there should be a balance between knowledge, understanding, and insight between the care provider and the patient. 

When a patient's insight of illnesses, knowledge, behavior, and preferences do not agree with the plan of care, choice of medication, route of administration, or problems of acceptance or compliance may arise. Disagreements often occur due to conscious personal choices, but sometimes disagreements result from an unconscious pathological condition known as “lack of insight.” Lack of insight usually complicates psychiatric presentations, requiring the assistance of a qualified medical professional, usually a psychiatrist, to determine the best plan of care. Lack of insight has been defined in various models but generally refers to the inability of a patient to understand and perceive the actual and accurate purpose behind his disease process or plan of care [5-7]. Lack of insight is seen as an obstacle to adequate medication administration and treatment because people with a lack of insight do not show responsible healthy behavior [6]. They may miss their medication, or appointments, show non-adherence to medicines, or [7] completely refuse pharmacotherapy [3]. Cohen et al. (2022) found that people with chronic mental health problems, chronic diseases that go along with mental health problems, disorders of cognition in children, and a cultural or low educational background often lack insight [6]. In accordance with the ethical principles of medicine, beneficence provides the obligation for healthcare professionals to provide the most effective medical treatments [8]. Even when a lack of insight is diagnosed, medication services should be provided to patients who lack insight, in addition to other treatment modalities like insight-oriented psychotherapy aimed at educating them regarding, the nature of their illness, the need for treatment, the medications, treatment modalities for their diseases, and the consequences of non-treatment [5, 9-10]. The ultimate short-term goal, therefore, in managing patients with a lack of insight is to provide therapeutic benefit and overcome the lack of insight barrier as quickly as possible [5-7, 9-10]. Therefore, it is important to choose practical and appropriate medication administration techniques for people lacking the insight to achieve maximum medication benefits.

The objective of the study

Review and highlight different approaches to administering medication to patients who lack insight into psychiatric conditions.

Method of literature search

The research methodology adopted in this study is the literature search strategy, which is done by retrieving and reviewing the existing literature relevant to medication administration techniques for people with no insight. The literature search strategy in this study was done by accessing online databases, including PubMed, Google Scholar, and Medline between 2010 and 2022. Specific keywords were used, which include "medication administration," "medicine," “lack of insight," “psychiatric patients with no insights," “involuntary medication in psychiatry” and "techniques." Moreover, Boolean operators were also used between these keywords so that the search results could be focused and directed toward the research objective. This was followed by a PRISMA flow chart based on the inclusion and exclusion criteria established for this study to choose and use relevant and careful literature to obtain information. The inclusion and exclusion criteria for this study have been described below in Table 1. 

**Table 1:** Inclusion and exclusion criteria.

Inclusion criteria	Exclusion criteria
1. Literature relevant to the techniques or methods of medication administration for people with a lack of insight in psychiatry	Works of literature that were published in a language other than English
2. Human studies	Animal studies
3. Randomized clinical trials, meta-analysis, practice guidelines, primary, secondary, or review studies relevant to medication administration and lack of insight in humans	Opinion pieces and non-scholarly articles
4. Works of literature published in English	Works of literature published other than in English (or English translation)
5. Works of literature published within the last 12 years (2010–2022)	

Search results

**Figure 1:**
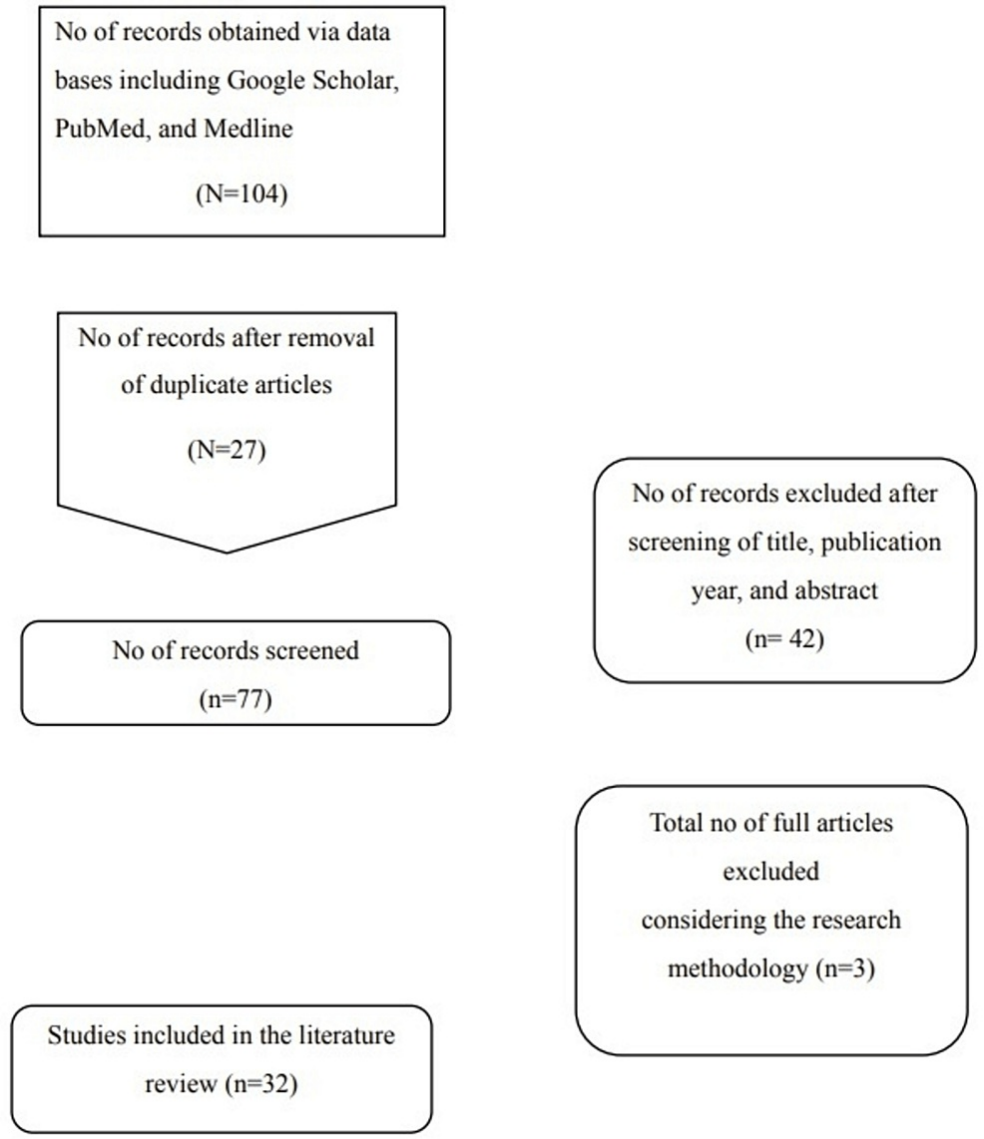
Study PRISMA diagram.

The initial search through PubMed, Google Scholar, and Medline retrieved 104 studies, among which 27 studies were found to have duplicated results. The remaining results were then further screened to review their titles and abstracts. Therefore, a careful review of the titles of the search results, publication year, and abstracts of the search results was done to determine the relevance of the search results concerning the research objectives. Out of 77, 42 studies were discarded due to the irrelevancy of the titles and abstracts because the study aimed to identify medication administration techniques for people with no insights. As described in the inclusion criteria, the search results within the 12 years were included, and the studies older than 2010 were excluded or discarded from the search results. This resulted in the shortlisting of 32 studies, and the researcher reviewed the copies of the full texts of the remaining studies. This full-text review helped identify the methodological approach of the studies, as this study is not focused on a particular research methodology. Moreover, the studies focused on medication administration techniques for people with insights and an in-depth understanding of medications were discarded to maintain the review objectives of this study. Hence, 32 studies were selected to be reviewed in this literature review, as presented in Figure 1 below, which represents the PRISMA diagram to show the search results of the study.

## Review

An overview of medication administration techniques in psychiatry

Different routes or administration methods are used depending on the nature of the medication or the desired medical effect. For example, injectables may have a desired faster onset of action. Other options may be slower. Oral, inhalational, transdermal, intrathecal, and suppositories have specific indications and onset of action [4, 11].

A medical overview of lack of insights

People often use the terms "lack of insight" and "no insight" interchangeably. A lack of insight characterizes several psychiatric illnesses. Several authors have proposed the definition of insight and its lack thereof, and these several models have evolved over the years [9-10]. David et al. [7] looked at several monomodal and multimodal descriptions of lack of insight. They came up with three co-dependent definitions: "the lack of recognition of having a mental illness, the lack of compliance with treatment, and the inability to label unusual mental events (like hallucinations) as pathological." The challenges physicians face during illness treatment due to lack of insight cannot be underestimated. One is a lack of medication adherence [5-7, 9-10, 12-13] and the consequent delay in the resolution of symptoms or illness. Several approaches to overcoming this challenge in psychiatry are evolving, and the already existing comprehensive intervention programs have been adopted locally or conventionally in different clinical settings. 

Lack of insight is present in various psychiatric disorders [5, 7, 9-10, 12], most common of which are found in schizophrenia [12-14] spectrum and psychotic disorders, bipolar and its spectrum, depression and its related illnesses, neurocognitive disorders, feeding and eating disorders, substance abuse, and some other mental disorders that do not fall into the traditional main types of psychiatric disorders. Insight and lack thereof make initiating therapy in patients difficult, and compliance becomes an overbearing obstacle when management is initiated. With poor compliance, symptom resolution is delayed, increasing hospitalization and the burden of these diseases [5, 7, 9-10, 12]. 

 Çetin and Aylaz (2018) performed an experimental controlled group design with pre-test and post-test control groups on a population of 369 patients with schizophrenia [3]. It was found that lack of insight among patients usually refers to patients with psychological disorders that could be preventive and normative under certain circumstances. This study suggests that people without insight need operational support to maintain hope, face a poor prognosis, or increase patient cooperation. Furthermore, people with no insight can be difficult for healthcare professionals, especially when they refuse medication due to a lack of understanding of the need for medical treatment [5, 7, 9-10,12-14].

A review article published by Lysaker et al. (2018) viewed insight among schizophrenia patients as a widespread psychological mechanism at the level of perception and adjustment along with the human experience of the disease [5]. They hinted that a lack of understanding about the importance of medication is a dynamic and multidimensional feature that develops from the initial symptoms of a lack of insight. Also, Cohen et al. (2022) conducted a mirror-image clinical trial that enrolled 113 schizophrenic adult patients between April 29, 2019, and August 11, 2020 [6]. They described cognitive deficits and behavioral patterns that cause a lack of insight. They claimed that more than half of patients with no insight suffer from psychiatric disorders, namely schizophrenia and bipolar disorder [6]. Furthermore, it was illustrated that there is growing evidence that lack of insight may be due to neurological deficits unrelated to the disease [6, 15]. It has been recognized that the pathophysiological causes of lack of insight in schizophrenia have a neuropsychological basis associated with anomalies in the frontal and temporal lobes of the brain [5, 7, 9-10, 12-14]. 

Pinho et al. (2021) studied the Beck Cognitive Insight Scale (BCIS) by recruiting 150 people with psychotic disorders who did not have insight [16]. This 15-item self-reported questionnaire evaluated patients' self-reflectiveness and overconfidence in their interpretations of their experiences. They assessed their understanding of medication administration knowledge and response. It was discovered that the cognitive processes of self-reflection, self-determination, high self-confidence, and low self-reflection had low scores and were reported to have poorer outcomes, understanding, and insight. It was also observed that people with a high score on the BCIS were associated with cooperation and involvement in the decision-making process for drug administration. One of the most prominent manifestations of lack of insight among patients is that they show strong negative reactions triggered by medication administration by healthcare professionals [12-14, 16]. It can also reflect a related pattern of impaired involuntary understanding caused by self-realization, self-esteem, and self-reliance disturbances [3, 5, 7, 9-10, 12-14]. However, we will discuss the challenges of the burden of lack of insight and medication administration, in our view, into emergency use administration and non-emergency conditions.

Emergency approaches to medication administration in patients who lack insight

The ethical dilemma in emergency psychiatry continues to remain debatable [8-17]. The general principle of autonomy, which permits patients to refuse or accept treatment, is frequently called into question when patients present with presentations that are clouded by impaired decision-making capacity and presenting with common symptoms like acute psychomotor agitation, suicidal ideations, homicidal ideations, threats of self-harm or violence to caretakers, and self-neglect [17-18]. These potentially fatal clinical symptoms can be seen in various common psychiatric illnesses, needing rapid treatment following a risk assessment [3, 5, 7, 9-10, 12-14]. The optimal course of treatment can be given to patients with these symptoms within 72 h of being detained against their will for a psychiatric examination under specific statutes in the United States. These guidelines, regulating laws, and approaches vary from state to state, and the providers who practice in each state or geographical region face unique difficulties. For instance, the Baker Act Regulation of Florida is a medicolegal-related regulation that permits the treating physician to detain and examine individuals who exhibit mental illness and are dangerous to themselves or others, including the risk of self-neglect and physical injury [19-20]. In other circumstances, medical professionals and associated caregiving services may keep a patient against their will after receiving permission from the relevant court [18-24]. After the assessment and work-up are finished, if the loss of insight is confirmed with other symptoms like impaired decision-making capacity, the necessity for hospitalization or other emergency psychiatric problems, the most expedient form of effective pharmacotherapy may be used. Because of their ease of administration, quick effect, and immediate objective of symptomatic alleviation, intramuscular (IM) and intravenous (IV) are frequently the most preferred routes in an acute care context, but typically only after attempts at situational soothing and confidence-building have failed [18-24].

Non-acute and maintenance approaches

Optimizing and simplifying pharmacologic regimens and addressing other strategies influencing patients' medication adherence like psychoeducational and behavioral techniques play a vital role in overcoming the challenges of lack of insight [22]. The non-acute phase of psychiatric disorders and their variations in lack of insight may require peculiarity in medication technique. The approaches to Schizophrenia Spectrum and Psychotic disorders may have their unique peculiarities [5, 7, 9-10, 12-14, 22]. The challenge of insight is evident during acute presentations of psychotic disorders and maintenance. Lack of insight is significantly seen in acute schizophrenic disorder [14], acute psychosis, and the manic phase of bipolar disorder. Given the varied ethical considerations on insight, the variety and lack of uniformly accepted standardized tools to measure insight, and the gravity of its role in treatment adherence, there seems to be a low threshold for defining lack of insight in the various clinical settings and after that, adopting a patient-centered treatment model that will overcome treatment delays in the best judgment of the managing team. Restraint and "forced injectables" may be an option when verbal engagement, non-pharmacologic approaches, and non-cohesive de-escalation techniques have failed [23] with some newer approaches of non-invasive methods like inhalational or rapid action oral onset agents when pharmacologic interventions are necessary. However, its ethical acceptance is still debatable [8, 12, 16-17]. In these acute phases, antipsychotics have been the go-to medications and can be administered in various forms. These antipsychotic injectables can be classified as short-acting or long-acting. In recent years, the use of long-acting injectables has been seen to help overcome this complex relationship of lack of insight, the different types and presentations of adherence, and then disease relapse [24]. Furthermore, the medication approach to lack of insight into mood disorders and their spectrum can equally be challenging. The notion of insight has been described in so many ways, even dependent on psychopathology. However, insight in a complex multidimensional model has three key components: awareness of the illness, labeling the current symptoms as part of the illness, and recognizing the importance of treatments [24-25]. Manic patients tend to have a severe lack of insight compared to patients with depression, who appear to have preserved insight even in acute episodes [22-25]. Lack of insight does play a critical role in treatment adherence for these patients, thus impacting their overall clinical outcome [22-25]. Although there is not much distinction in the medication approach for these patients who lack insight, improving it could improve outcomes. Some studies have noted that treatment-alliance between clinicians and patients has mitigated the issue of non-adherence to medications [8, 12, 16, 26], thereby gradually improving the insights of these patients. Other studies have emphasized the influence of patients' families on medication non-adherence [24, 27], stating that dysfunctional families are associated with poor medication adherence. Therefore, a more functional family environment could breed stronger medication adherence among these patients. Similarly, the approach in cases of substance abuse and lack of insight, literature shows that many individuals with substance abuse fail to seek treatment and live in denial [15, 28]. This aftermath can be enormous, ranging from missed appointments, repeated hospitalization, and medication administration without the patient's consent [29]. Counseling, cognitive behavioral therapy, and sometimes surreptitious administration [30] are sometimes deployed. However, the potential therapeutic problems associated with crushing prolonged-release tablets or capsules are well understood. Although, administering medicines through fruit juices (grapefruit, apple, or orange juice) interferes with the absorption of some medications, mainly due to the phenotypic expression of Phase I and Phase II drug metabolism especially the CYP3A4 enzyme which is expressed mainly in the small intestine and liver [31]. Other times, medications are administered involuntarily, involving parenteral injections to restore physiologic function substantially. In the principle of medical ethics, a psychiatrist may only authorize the use of their certification for the involuntary treatment of any person after having personally examined that person [8, 10, 16, 17-19]. To do so, the physician must determine that the person's mental condition prevents them from deciding what is in their best interests and that there is a significant risk to them or others in the absence of treatment. Several patient-rights groups have criticized the medico-legal factor that underpins this strategy [32]. A 2003 Supreme Court decision in Sell v. United States (539 U.S. 166) established a new standard [33-34]. As a result of their retrospective record review of all incompetent defendants in the U.S. federal court system (N = 132) who were involuntarily treated under Sell over six years, Robert and his team concluded that the majority (79%) of treated defendants with a psychotic related illness were sufficiently improved to be made competent to stand trial, exceeding the "clear and convincing" standard set by federal appellate courts. Although various clinical, ethical, and privacy rights have been made against this strategy. Kirk et al. also emphasized that extended hospitalization, in addition to the perceived ethical violation, could be a barrier to this technique [32-34]. Patients will benefit, however, from efforts to create efficient medicine delivery systems in cases with a lack of insight. 

Technology advancements may have their own role [6]. More recently, the possibility of using technology to alleviate the burden of lack of insight has been explored. These advances affect modes of delivery, intervals of medication delivery, pharmacodynamics, and kinetics of disease-specific disorder needs in psychiatry. For example, Abilify MyCite, a recent technology aimed at overcoming some of the problems of lack of insight, medication compliance, and decision-making capacity, has been explored. Klein et al. (2021) conducted a cross-sectional study to identify the effectiveness of human technologies that are used to affect the human psyche [35]. ABILIFY MYCITE is a prescribed aripiprazole tablet with an ingestible event marker (IEM) sensor designed to treat schizophrenia, bipolar disease alone or with lithium or valproate, acute or short-term treatment of anxiety or manic symptoms, palliative care, and treatment of depression. The sensor is swallowed and sends a signal to give real-time data and information on the pharmacokinetics and kinetics of its content. However, treatment of these psychological disorders is combined with other medications as the primary goal of this drug is to track the medication data. The system checks for medication compliance. This is especially important for people at risk of forgetting or having complex mental health disorders. Another example of technological advancement can be seen in 3D-printed drug technology. This process was patented in 1986 but has only become popular in the last 10 years. 3D-printing (3DP) research on medical applications for bio-printing, prosthetics, and pharmaceuticals is rising. The new method produces customized dosage forms with variable geometry and dimensions. This changes how the product is released and how it is given to the body. However, 3DP creates the potential for personalized medication systems that are independent of the ability of the patient to comply with treatment, for instance, an ultra-long-acting controlled release delivery system can be developed to deliver constant daily doses of antipsychotic medication for a year. This bypasses the patient's effort, effectively avoiding non-compliance due to a lack of insight. Lepowsky and Tasoglu (2018) [36] while reviewing the advances made in 3DP, gave examples of the types of 3DP, namely selective laser sintering, stereolithography, binder deposition, extrusion printing, inkjet printing, and fused deposition modeling. This may be a big step forward in medication technology. These new methods produce customized dosage forms with variable geometry and dimensions [35-36]. These developments open up fresh avenues for investigating how to get around the intricate connection between medication administration and lack of insight.

Study limitation

A notable limitation of this literature review is the personalized appraisal method by the different co-authors who reviewed segments of the various articles. This is consistent with the well-known problem of standardization in systematic reviews and the use of a uniformly standardized validation scale in evaluating selected pieces of literature in a literature review. Additionally, selection bias may have played a role. While reviewing scholarly articles that meet the inclusion criteria (see Appendix), we may have inadvertently overlooked other relevant literature pertaining to the purpose of this study that may be accessible from other scholarly repositories or may have been titled in a way that evaded our search strategy or study design.

## Conclusions

Administering medication to patients who lack insight can be challenging. However, there has been some advancement to mitigate this barrier. Common moral values, common sense, medicolegal support, person-centered integrated care, and innovative medication techniques may play a role. Overcoming the obstacles of lack of insight into psychiatric disorders has proven helpful in the overall prognosis or outcome of psychiatric diseases. However, these models of medication administration are still evolving, with their attendant ethical concerns. Hopefully, the available models discussed in this review will provide the base rock for future advancements. There is still a lot to be done. We encourage modern-day research to explore safer and more dynamic approaches that can mitigate this condition.

## Appendices

**Table 2:** Pieces of literature that met the final inclusion criteria and that were included in this study.

	Enoch (2019) [1]
	McMahon (2014) [2]
	Çetin and Aylaz (2018) [3]
	Melo et al. (2021) [4]
	Lysaker et al. (2018) [5]
	Cohen et al. (2022) [6]
	David et al. (2012) [7]
	American Psychiatric Association, 2013 edition, (2013) [8]
	Murri and Amore (2019) [9]
	Reddy (2016) [10]
	Correll et al. (2021) [11]
	Konstantakopoulos (2019) [12]
	Novick et al. (2015) [13]
	Varga et al. (2007) [14]
	Dean et al. (2015) [15]
	Pinho et al. (2021) [16]
	Allen et al. (2015) [17]
	Sessums et al. (2011) [18]
	Maniaci et al. (2019) [19]
	Baker Act Involuntary Examination Criteria (2022) [20]
	Mavrogiorgou et al. (2011) [21]
	Phan (2016) [22]
	Zeller and Citrome (2016) [23]
	Buchman-Wildbaum et al. (2020) [24]
	Crișan (2018) [25]
	Cely et al. (2011) [27]
	Goldstein et al. (2009) [28]
	Williams et al. (2015) [29]
	Latha and Phil (2010) [30]
	Cochrane et al. (2018) [33]
	Klein et al. (2021) [35]
	Lepowsky and Tasoglu (2017) [36]

## References

[1] Enoch RG (2019). Therapeutic modalities in psychiatry. Slideshare.

[2] McMahon FJ (2014). Prediction of treatment outcomes in psychiatry--where do we stand ?. Dialogues Clin Neurosci.

[3] Çetin N, Aylaz R (2018). The effect of mindfulness-based psychoeducation on insight and medication adherence of schizophrenia patients. Arch Psychiatr Nurs.

[4] Melo GB, Cruz NF, Emerson GG (2021). Critical analysis of techniques and materials used in devices, syringes, and needles used for intravitreal injections. Prog Retin Eye Res.

[5] Lysaker PH, Pattison ML, Leonhardt BL, Phelps S, Vohs JL (2018). Insight in schizophrenia spectrum disorders: relationship with behavior, mood and perceived quality of life, underlying causes and emerging treatments. World Psychiatry.

[6] Cohen EA, Skubiak T, Hadzi Boskovic D (2022). Phase 3b multicenter, prospective, open-label trial to evaluate the effects of a digital medicine system on inpatient psychiatric hospitalization rates for adults with schizophrenia. J Clin Psychiatry.

[7] David AS, Bedford N, Wiffen B, Gilleen J (2012). Failures of metacognition and lack of insight in neuropsychiatric disorders. Philos Trans R Soc Lond B Biol Sci.

[8] American Psychiatric Association (2013). The Principles of Medical Ethics. https://www.psychiatry.org/File%20Library/Psychiatrists/Practice/Ethics/principles-medical-ethics.pdf.

[9] Belvederi Murri M, Amore M (2018). The multiple dimensions of insight in schizophrenia-spectrum disorders. Schizophr Bull.

[10] Reddy MS (2016). Lack of insight in psychiatric illness: a critical appraisal. Indian J Psychol Med.

[11] Correll CU, Kim E, Sliwa JK (2021). Pharmacokinetic characteristics of long-acting injectable antipsychotics for schizophrenia: an overview. CNS Drugs.

[12] Konstantakopoulos G (2019). Insight across mental disorders: a multifaceted metacognitive phenomenon. Q J Hellenic Psychiatric Assoc.

[13] Novick D, Montgomery W, Treuer T, Aguado J, Kraemer S, Haro JM (2015). Relationship of insight with medication adherence and the impact on outcomes in patients with schizophrenia and bipolar disorder: results from a 1-year European outpatient observational study. BMC Psychiatry.

[14] Varga M, Magnusson A, Flekkøy K, David AS, Opjordsmoen S (2007). Clinical and neuropsychological correlates of insight in schizophrenia and bipolar I disorder: does diagnosis matter?. Compr Psychiatry.

[15] Dean AC, Kohno M, Morales AM, Ghahremani DG, London ED (2015). Denial in methamphetamine users: associations with cognition and functional connectivity in brain. Drug Alcohol Depend.

[16] Pinho LG, Sampaio F, Sequeira C, Martins T, Ferré-Grau C (2021). Cognitive insight in psychotic patients institutionalized and living in the community: an examination using the Beck Cognitive Insight Scale. Psychiatry Res.

[17] Allen NG, Khan JS, Alzahri MS, Stolar AG (2015). Ethical issues in emergency psychiatry. Emerg Med Clin North Am.

[18] Sessums LL, Zembrzuska H, Jackson JL (2011). Does this patient have medical decision-making capacity?. JAMA.

[19] Maniaci MJ, Burton MC, Lachner C (2019). Patients threatening harm to others evaluated in the ED under the Florida Involuntary Hold Act (Baker Act). South Med J.

[20] (2022). Baker Act 
Involuntary Examination
Criteria, Processes and Timeframes. https://www.usf.edu/cbcs/baker-act/documents/bakeractcriteriaprocesses.pdf.

[21] Mavrogiorgou P, Brüne M, Juckel G (2011). The management of psychiatric emergencies. Dtsch Arztebl Int.

[22] Phan SV (2016). Medication adherence in patients with schizophrenia. Int J Psychiatry Med.

[23] Zeller SL, Citrome L (2016). Managing agitation associated with schizophrenia and bipolar disorder in the emergency setting. West J Emerg Med.

[24] Buchman-Wildbaum T, Váradi E, Schmelowszky Á, Griffiths MD, Demetrovics Z, Urbán R (2020). The paradoxical role of insight in mental illness: The experience of stigma and shame in schizophrenia, mood disorders, and anxiety disorders. Arch Psychiatr Nurs.

[25] Crișan CA (2018). Lack of insight in bipolar disorder: the impact on treatment adherence, adverse clinical outcomes and quality of life. IntechOpen.

[26] Martin DJ, Garske JP, Davis MK (2000). Relation of the therapeutic alliance with outcome and other variables: a meta-analytic review. J Consult Clin Psychol.

[27] Cely EE, Fierro M, Pinilla MI (2011). Prevalence and associated factors of non-adherence to treatment in bipolar disorder. Res Gate.

[28] Goldstein RZ, Craig AD, Bechara A, Garavan H, Childress AR, Paulus MP, Volkow ND (2009). The neurocircuitry of impaired insight in drug addiction. Trends Cogn Sci.

[29] Williams AR, Olfson M, Galanter M (2015). Assessing and improving clinical insight among patients “in Denial”. JAMA Psychiatry.

[30] Latha KS (2010). The noncompliant patient in psychiatry: the case for and against covert/surreptitious medication. Mens Sana Monogr.

[31] Pawełczyk T, Kłoszewska I (2008). [Grapefruit juice interactions with psychotropic drugs: advantages and potential risk]. Przegl Lek.

[32] (2022). Forced medication and competency to stand trial: clinical, legal, and ethical issues. https://www.psychiatrictimes.com/view/forced-medication-and-competency-stand-trial-clinical-legal-and-ethical-issues.

[33] Cochrane RE, Herbel BL, Reardon ML, Lloyd KP (2013). The Sell effect: involuntary medication treatment is a "clear and convincing" success. Law Hum Behav.

[34] (2022). Sell v. United States, 539 U.S. 166 (2003). https://supreme.justia.com/cases/federal/us/539/166/.

[35] Klein E, Mills C, Achuthan A (2021). Human technologies, affect and the global psy-complex. Econ Soc.

[36] Lepowsky E, Tasoglu S (2018). 3D printing for drug manufacturing: a perspective on the future of pharmaceuticals. Int J Bioprint.

